# Self-assembly of “patchy” nanoparticles: a versatile approach to functional hierarchical materials

**DOI:** 10.1039/c5sc01141h

**Published:** 2015-05-12

**Authors:** David J. Lunn, John R. Finnegan, Ian Manners

**Affiliations:** a School of Chemistry , University of Bristol , Bristol BS8 1TS , UK . Email: ian.manners@bristol.ac.uk

## Abstract

The solution-phase self-assembly or “polymerization” of discrete colloidal building blocks, such as “patchy” nanoparticles and multicompartment micelles, is attracting growing attention with respect to the creation of complex hierarchical materials. This approach represents a versatile method with which to transfer functionality at the molecular level to the nano- and microscale, and is often accompanied by the emergence of new material properties. In this perspective we highlight selected recent examples of the self-assembly of anisotropic nanoparticles which exploit directional interactions introduced through their shape or surface chemistry to afford a variety of hierarchical materials. We focus in particular on the solution self-assembly of block copolymers as a means to prepare multicompartment or “patchy” micelles. Due to their potential for synthetic modification, these constructs represent highly tuneable building blocks for the fabrication of a wide variety of functional assemblies.

## Abstract 2

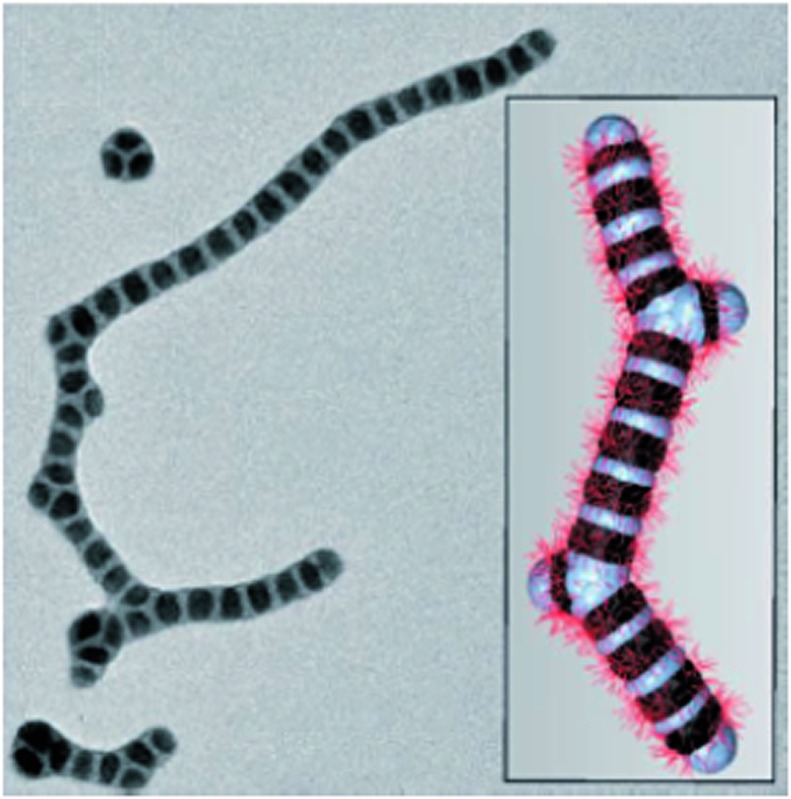
The solution-phase self-assembly or “polymerization” of discrete colloidal building blocks, such as “patchy” nanoparticles and multicompartment micelles, is attracting growing attention with respect to the creation of complex hierarchical materials.

## 1 Introduction

“Top-down” and “bottom-up” are two well-known, complementary, and all-encompassing design strategies for the preparation of functional nanomaterials. Top-down approaches focus either on the breaking down of a pre-existing hierarchical system into its constituent parts, or on the guided transfer of order during material preparation. Examples include the deconstruction of a virus and isolation of its capsid for biomedical applications, and the template directed reactive-ion-etching of bulk silicon to prepare microchips bearing nanometre-scale features. Bottom-up approaches focus on the assembly of subunits or ‘building blocks’ to prepare increasingly complex and hierarchical systems, categorized as either directed assembly or self-assembly. In directed assembly, the formation of subunits is guided by external stimuli,^1^ for example by magnetic fields^2^ or optical tweezers.^3^ In contrast, self-assembly exploits the predefined interactions of discrete subunits to prepare organized structures.^4^,^5^ To maintain order throughout hierarchical assemblies, which span length scales orders of magnitude greater than the feature sizes of the building blocks from which they are constructed, each subunit must be well-defined and able to combine with others in a controlled manner.

Despite the bewildering complexity achieved in Nature through bottom-up self-assembly, the actual pool of molecular building blocks used is relatively small. Starting with a simple collection of just over 20 amino acids, 5 nucleotides, a handful of sugars and a series of abundant metal ions, structures such as polypeptides, nucleic acids, polysaccharides and hybrid organic–inorganic materials (*e.g.* bone, with many levels of structural hierarchy) can be prepared.^6^ Complexity arises from the self-assembly of these building blocks using a variety of molecular interactions, for example electrostatic, hydrogen bonding, metal–ligand and π-donor/acceptor, as well as many other covalent and non-covalent linkages.^7^ Polypeptides prepared by the chain growth polymerization of amino acid subunits by the translation of messenger ribonucleic acid (mRNA), or within pre-existing enzymes, undergo intramolecular chain folding through non-covalent interactions determined by the sequence of amino acids. This chain folding affords secondary structures such as α-helices, β-sheets and β-turns, which drive the further self-assembly of the polypeptide into more complex tertiary structures. These tertiary structures can constitute the highest level of structural order for a single protein, or alternatively, can function as subunits for the further growth of hierarchical assemblies. For example, the globular multifunctional protein actin can undergo controlled self-assembly or “polymerization” to form quaternary structures in the form of filaments, which are responsible for a variety of crucial structural functions in the cell.^8^ As disorder in the resulting assemblies may lead to biomaterial failure or cell death, Nature maximizes the efficiency of self-assembly by preparing low energy structures.^7^,^9^ Using dynamic molecular interactions that have some degree of reversibility, the resulting self-assembled structures have the ability to error-check and self-repair.^7^,^10^,^11^

**Fig. 1:**
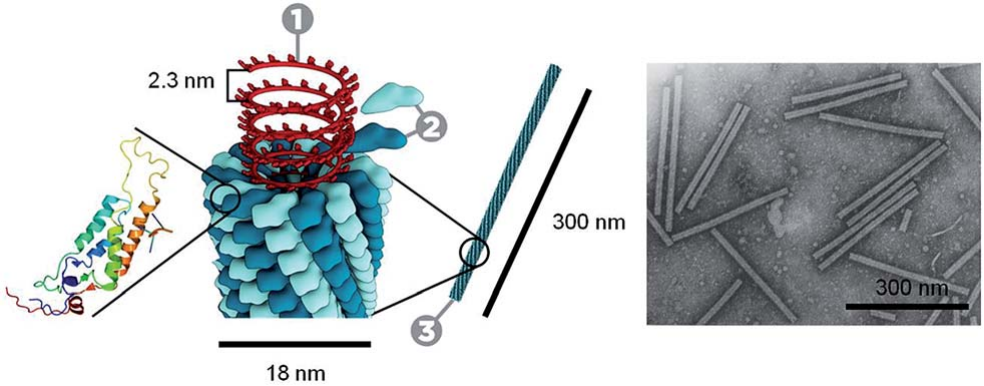
Schematic representation and TEM image of the tobacco mosaic virus. One molecule of single strand RNA (1) surrounded by 2130 coat proteins (2) that make up the virus capsid (3). Transmission electron microscopy (TEM) image©1994 Rothamsted Experimental Station. Tobacco mosaic schematic by Thomas Splettstoesser (http://www.scistyle.com).

Through the self-assembly of well-defined subunits, the information required to encode hierarchical systems is minimized. The tobacco mosaic virus (TMV), the first to be discovered and considered the herald of modern virology, is comprised of 2130 identical protein subunits each containing 158 amino acids. These subunits self-assemble around a single strand of RNA to form a stable structure 300 nm long (Fig. 1). If genetic information were required to encode the entire structure, the genome would be larger than the virus itself. However, to encode the identical subunits that undergo defined self-assembly requires only a small percentage of the total RNA.^12^

TMV illustrates how Nature achieves hierarchical self-assembly so elegantly to create functional materials with many levels of substructure.^6^,^9^,^10^ Considerable effort is still focussed on understanding natural self-assembly processes based on the idea that new knowledge and insight can be harnessed for the creation of synthetic routes to novel structures with reduced defects and enhanced properties. As methods for the preparation of “patchy” particles have improved, a large variety of new building blocks has become widely accessible. In this Perspective we have sought to highlight a selection of recent examples, wherein the relatively weak but directional interactions between “patchy” particles are exploited to prepare hierarchical assemblies. First, we discuss supramolecular polymerizations, which provide a conceptual foundation for the self-assembly processes described in later discussions.

## 2 Supramolecular polymerizations *via* directional non-covalent interactions

Supramolecular polymerization under thermodynamic control refers to the self-assembly of molecular monomers by “moderately strong, reversible non-covalent, but highly directional forces that result in high molecular weight linear polymers under dilute conditions”.^13^ Whereas a conventional covalent polymer is considered to be in a “static” state due to the non-reversibility of the covalent bonds between monomeric units, a supramolecular polymer formed by a process of this type will remain in a state of dynamic equilibrium. Nevertheless, despite their intrinsic dynamic structure, supramolecular polymers have been prepared that display mechanical properties in the bulk that were believed to be achievable only in covalently bonded polymeric materials. Moreover, on mild thermal treatment, easily processed, low viscosity materials result, which is an advantage for many applications.^14^

Supramolecular polymers can exploit a variety of weak intermolecular forces (*e.g.* hydrogen bonding, π-stacking/crystallization, metallophilic or hydrophobic/hydrophilic interactions). The resulting structure and function can be tuned by synthetic modification of the starting monomers, which can consist of molecules and also larger subunits such as proteins (as in TMV and natural filaments such as F-actin and microtubules). By designing monomers for a specific purpose, supermolecular polymers with useful mechanical, biological, optical and electronic functionality can be prepared.^15^ The lifetime of the interactions responsible for self-assembly can also be controlled by synthetic modification of the monomer subunits. Through lifetime control, dynamic systems can be prepared that are self-repairing, adaptive and responsive to external stimuli. Furthermore, by modifying the valency of constituent monomer building blocks, 2D and 3D networks and arrays could be prepared. For example, the self-assembly of triblock oligomers into 2D supramolecular monolayers and films has been described where formation of the 200 kilodalton mushroom-shaped subunits is believed to be driven by the crystallization of a core segment based on biphenyl ester units through π–π interactions.^16^

The mechanisms of supramolecular polymerizations can be broadly classified into two types: isodesmic and nucleation–elongation/cooperative polymerizations.^13^,^17^ In the isodesmic case, the equilibrium constants associated with the successive addition of each monomer are equal. This is analogous to a step growth covalent polymerization of molecular monomers. In contrast, for a nucleation–elongation process the analogous equilibrium constants are not equal, more commonly possessing a smaller value up to a nucleation event and a larger one for the subsequent elongation steps.

A more recent area of focus for supramolecular polymers involves the study of systems that proceed under kinetic rather than thermodynamic control.^13^,^18^ Although relatively weak and directional non-covalent interactions also bind the monomeric units together in this case, the resulting materials exist in a non-equilibrium, metastable state. Dynamic behavior is switched off as the activation energy barrier for exchange, while still low, is large relative to thermal energies (*kT*). Kinetically-trapped supramolecular polymers can offer intriguing features that differ significantly from their dynamic analogs. For example, “living” supramolecular polymerizations have been recently achieved in the case of nucleation–elongation processes by the use of seeded growth methods and, in many ways, these are analogous to living chain growth covalent polymerizations. Thus, kinetically-trapped supramolecular polymers can be formed that are monodisperse in length and can even possess segmented or block architectures. The formation of such materials has recently been achieved in the cases of molecularly dissolved block copolymer building blocks that form fiber-like micelles with a 1D crystalline core (see Section 4.2),^19^–^23^ and also with hexabenzocoronene^24^ and metalloporphyrin^25^ derivatives that, through π-stacking interactions, generate 1D nanotubes and nanofibers, respectively. Analogous future developments for other stacked assemblies, such as perylene diimides for example, appear highly likely.^26^–^28^ In another fascinating example, monodisperse supramolecular polymers based on hydrogen bonding interactions between corannulene molecules have been prepared by the treatment of metastable monomers with appropriate initiators.^29^

As a result of their pervasive significance, the fundamental understanding of supramolecular polymerizations that proceed under either thermodynamic or kinetic control provides an inspirational conceptual foundation with respect to the use of weak interactions for the self-assembly of multicompartment or “patchy” nanoparticles described in the following sections.

## 3 Directional self-assembly of “patchy” nanoparticles

The preparation of organized assemblies of colloidal nanoparticles is of particular interest as they often display different properties to those of their component subunits. For example, metal nanoparticle assemblies can exhibit electrical,^30^ optical and magnetic^31^ properties that are distinct from those of the individual nanoparticles and the corresponding bulk material. Isotropic spherical particles can undergo self-assembly to form close-packed crystals. However, limited structural variety is possible for the resulting assemblies due to the lack of directional interactions. Self-assembly in Nature exploits the directional interactions of anisotropic subunits, through which assemblies with enormous morphological diversity can be prepared.

### 3.1 Janus-type “patchy” polystyrene particles

**Fig. 2:**
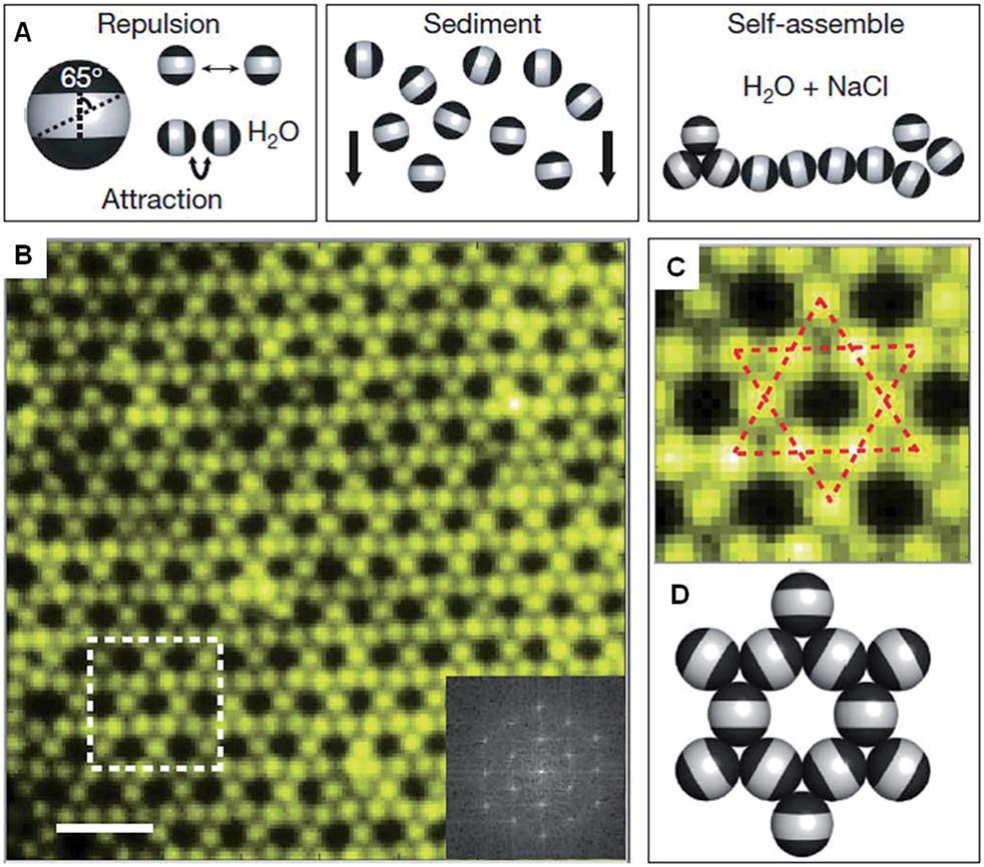
Colloidal kagome lattice after equilibration. (A) Triblock Janus particles, charged in the equator section (white) and made hydrophobic on the poles (black), are allowed to sediment in deionized water. NaCl is added to screen electrostatic repulsion, allowing self-assembly by short-range hydrophobic attraction. (B) Fluorescence image of a colloidal kagome lattice and its fast Fourier transform image (bottom right). Scale bar corresponds to 4 μm. (C) Enlarged view of the dashed white rectangle in (B). Dotted red lines highlight two staggered triangles. (D) Schematic illustration of particle orientations. Reproduced with permission from ref. 32.

Recent work has shown that through the selective patterning of hydrophobic poles onto spherical, negatively charged polystyrene particles, multicompartment subunits can be prepared that undergo directional self-assembly to afford well-defined lattices (Fig. 2).^32^ The starting triblock Janus particles were readily prepared by selectively gold-plating sulphate polystyrene spheres and made hydrophobic through the deposition of *n*-octadecanethiol monolayers.

**Fig. 3:**
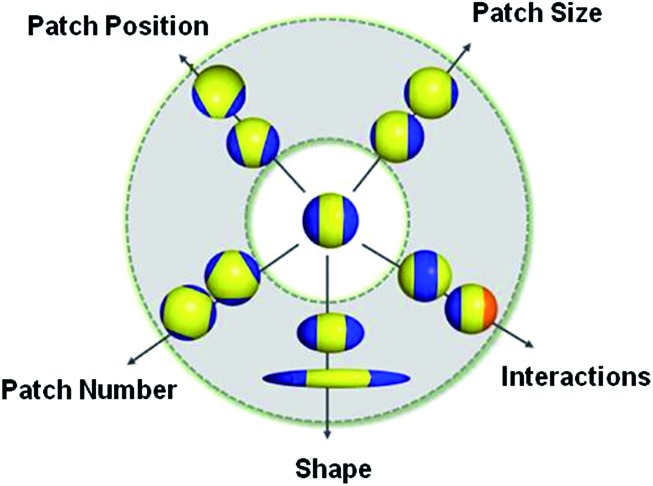
Potential routes for the modification of “patchy” colloidal particles. Blue and orange regions represent patches of selectively located chemical functionality. These parameters are also applicable to other shapes such as rods, cubes, and other polyhedra. Reproduced with permission from ref. 34. Copyright 2012 American Chemical Society.

This “patchy” particle self-assembly approach was extended to prepare colloidal structures displaying hierarchical order by the stepwise assembly of particles with different sized hydrophobic domains.^33^ The directional interactions that govern such colloidal self-assembly can be further manipulated by changing the particle shape, as well as the number, position and chemistry of their surface patches (Fig. 3).^34^ For example, the reversible aggregation of Janus polystyrene particles has been achieved through the use of surface-grafted supramolecular moieties that exploit host–guest interactions.^35^

### 3.2 DNA-nanoparticle hybrid materials

The self-assembly of nanoparticles into pre-defined arrangements is a simple concept, but is more challenging to achieve in practice due to difficulties associated with their selective functionalization. Nanoparticle capping ligands that prevent uncontrolled aggregation can contain complex functionality and can be selectively localized. Using this strategy well-defined nanoparticle assemblies have been prepared by the self-assembly of gold nanoparticles selectively functionalized with specific nucleic acids. In 1996 it was shown that strands of DNA could be used to reversibly assemble spherical gold nanoparticles into macroscopic aggregates.^36^ This approach was extended to demonstrate that the DNA sequence could be used to define the crystalline state of the packed nanoparticles^37^ and that anisotropic building blocks could be employed.^38^ Furthermore, the development of a general method for the nucleic acid functionalization of nanoparticles allowed a variety of different elements and compounds to be used as building blocks for these assemblies.^39^ Complementary strategies have also shown that DNA itself can be used as a helical template for the directed assembly of nanoparticles.^40^,^41^

**Fig. 4:**
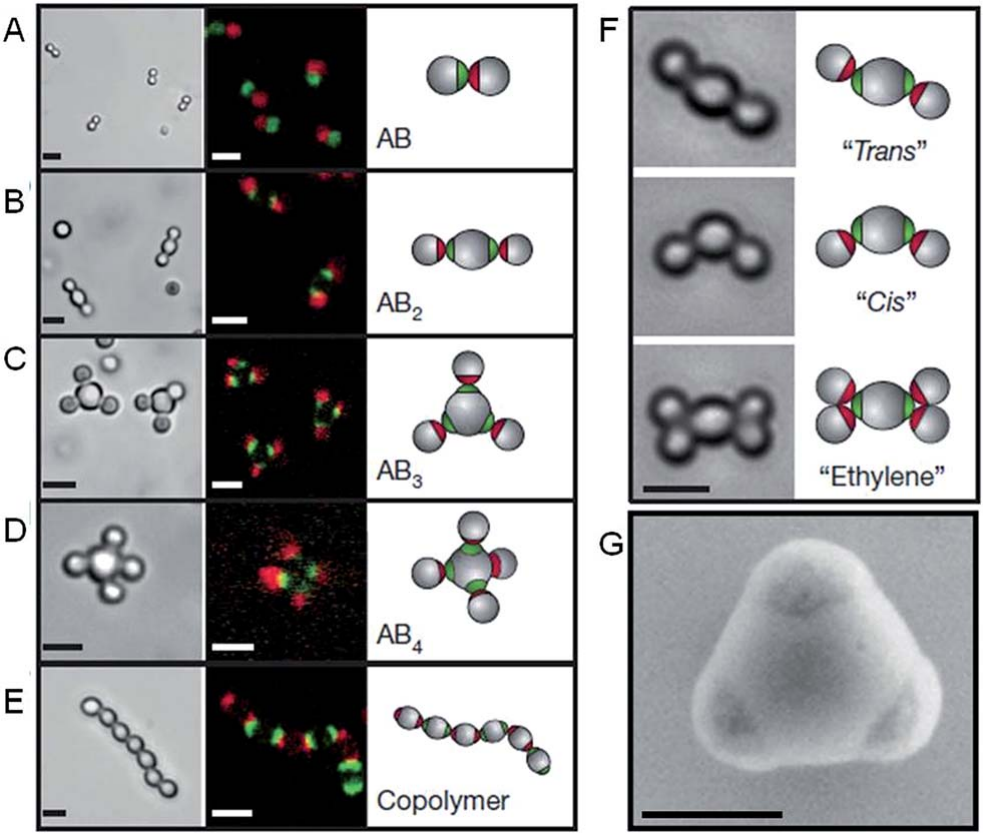
Colloidal molecules self-assembled by specific directional bonding between particles. Bright-field optical microscopy (left panels), confocal fluorescent (middle panels A–E) and schematic images (right panels). Scale bars correspond to 2 μm. (A) Complementary green and red monovalent particles form dumbbell-shaped AB-type molecules. Supracolloidal molecules AB_2_, AB_3_ and AB_4_ are formed by mixing red monovalent with green divalent (B), trivalent (C) and tetravalent (D) particles. (E) If complementary divalent particles are mixed, linear alternating polymer chains spontaneously assemble. (F) When particles with bigger patches are used, ‘cis’ and ‘trans’ isomers can form. Introducing more monovalent particles leads to colloidal ‘ethylene’ molecules. Scale bars correspond to 2 μm. (G) Scanning electron microscopy (SEM) image of trivalent patchy particle. Scale bar corresponds to 500 nm. Reproduced with permission from ref. 42.

Recent work has greatly increased the sophistication of possible self-assembled nanoparticle structures by using a similar DNA functionalization approach to prepare colloidal analogues of hybridized atoms.^42^ These colloidal particles, comprised of amidinated polystyrene and bearing chemically distinct surface patches functionalized with DNA, imitate hybridized atomic orbitals including sp, sp^2^, sp^3^, sp^3^d, sp^3^d^2^ and sp^3^d^3^ (Fig. 4). At the termini of the DNA are single-strand “sticky” ends, allowing these “atom equivalents” to form directional bonds through programmable, specific and reversible DNA interactions.

### 3.3 Polymer-nanoparticle hybrid materials

**Fig. 5:**
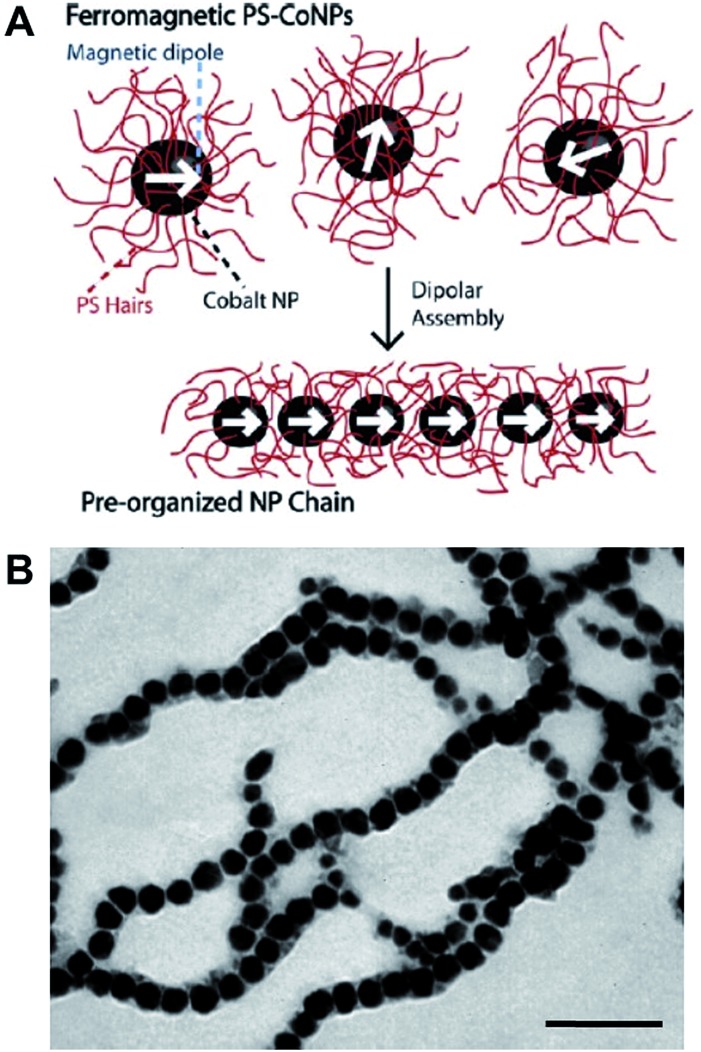
Dipolar self-assembly of ferromagnetic cobalt nanoparticles. (A) Schematic representation of the dipolar self-assembly of polystyrene-coated cobalt nanoparticles. (B) Representative TEM image of cobalt nanoparticle nanowires. Scale bar corresponds to 100 nm. Reproduced with permission from ref. 45. Copyright 2009 American Chemical Society.

Polymers can be used to direct, assist or stabilize the organization of otherwise disordered nanoparticle systems. Using approaches akin to traditional covalent polymerizations, monomeric nanoparticles can be self-assembled through step or chain growth type colloidal “polymerizations” into mesoscopic chains.^43^,^44^ For example, polystyrene-coated ferromagnetic cobalt nanoparticles have been shown to align into single line nanoparticle chains in the absence of a magnetic field due to interparticle dipolar associations (Fig. 5).^45^ This process has been termed dipolar self-assembly and has been used to prepare Au–Co_3_O_4_ and Pt–Co_3_O_4_ nanowires that showed enhanced electrochemical properties.^46^,^47^

**Fig. 6:**
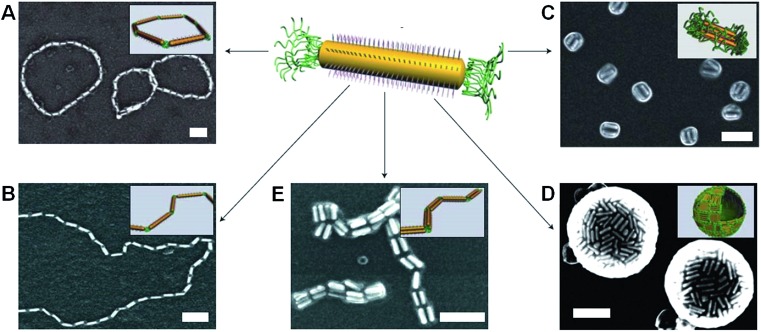
Self-assembly of polymer-tethered gold nanorods in selective solvents. An amphiphilic gold nanorod carrying a double layer of cetyl trimethylammonium bromide along the longitudinal side and polystyrene molecules grafted to both ends. SEM images of the self-assembled nanorod structures: rings (A), chains (B), side-to-side aggregated bundles (C), nanospheres (D) and bundled nanorod chains (E). Scale bars correspond to 100 nm. The insets show schematic diagrams for the nanorod assemblies. Reproduced with permission from ref. 48.

Metal-polymer analogues of amphiphilic triblock copolymers have been prepared by functionalizing the ends of stable hydrophilic gold nanorods with hydrophobic polystyrene.^48^,^49^ By changing the solvent composition, self-assembly could be induced to organize the nanorods into a variety of geometries, including rings, chains, aggregated bundles and larger superstructures (Fig. 6).

The self-assembly process that afforded chains and rings of gold nanorods was shown to be analogous to a step-growth polymerization, allowing quantitative predictions to be made about the aggregation number and size distributions of the resulting structures.^50^ In other work, block copolymer coated gold nanoparticles were observed to self-assemble into single- or double-line chains through the utilization of the sphere-to-cylinder transition of the polymers.^51^ In this approach the addition of a single nanoparticle to the growing chain was favoured over the aggregation of monomeric units, resulting in a nanoparticle self-assembly process analogous to a chain growth polymerization.

## 4 Hierarchical self-assembly of “patchy” block copolymer micelles

For the preparation of complex hierarchical structures by self-assembly, some form of anisotropy or directional interaction is required through the shape and/or surface functionality of the starting subunits.^52^ Monodisperse and well-defined “patchy” micelles, prepared by the solution self-assembly of amphiphilic block copolymers, represent ideal building blocks for the preparation of hierarchical structures. Due to improved methods for the rational design and synthesis of multiblock copolymers,^53^ a large variety of potential “patchy” micelles can be envisaged.

### 4.1 Preparation and stabilization of block copolymer micelles

In Nature, the polypeptide chains required for the preparation of a target protein are of the same length and composition, having a polydispersity index (PDI) of exactly 1. As protein function arises due to precise chain folding of these monodisperse polypeptides, an important target for polymer chemists is the preparation of monodisperse macromolecules that will minimize structural defects during self-assembly. A range of covalent polymerization methods that approximate to living processes^54^ have been developed for the preparation of block copolymers, including anionic,^55^ cationic,^56^ atom transfer radical polymerization (ATRP),^57^ reversible addition-fragmentation transfer (RAFT) polymerization,^58^ nitroxide-mediated radical polymerization^59^ and ring-opening metathesis polymerization (ROMP).^60^

The solution self-assembly of such amphiphilic block copolymers is arguably one of the most important methods for the preparation of well-defined and functional nanostructures.^61^,^62^ Above the critical micelle concentration (CMC) (or critical aggregation concentration), amphiphilic block copolymers will undergo self-assembly to minimize the unfavourable interactions of the insoluble block with the selective solvent. This aggregation results in an overall decrease in the free energy of the system by removing the solvophobic blocks from solution. In contrast to micelles formed by small surfactant molecules, block copolymer micelles generally exhibit slow or negligible intermicelle unimer exchange kinetics due to the high free energy cost associated with transferring a polymer with a long solvophobic block into solution. This means that block copolymer micelles can often be considered to be kinetically frozen.^63^ Block copolymer self-assembly in solution is complex and a variety of factors determine the resulting morphologies, including concentration, temperature, block copolymer composition and architecture, solvent composition and the presence of additives.^64^,^65^ An enormous variety of equilibrium and kinetically-trapped micelle morphologies are accessible,^65^ and this can be further enriched by the self-assembly of block copolymer blends. For example, the blending of bilayer-forming and cylinder-forming block copolymers has been used to access hybrid disk micelle morphologies.^66^ As the resulting micelles can be used to segregate and immobilize chemical functionality, they have found widespread use in a variety of applications,^67^ including as vessels for biomedical delivery^68^,^69^ and as catalytic nanoreactors.^70^,^71^

Block copolymer micelles are more robust than those formed by molecular surfactants. However, their potentially dynamic behaviour is a disadvantage when preparing structures that need to remain stable below the CMC, with changes in temperature, or on addition of a common solvent. If appropriate functionality is incorporated into the block copolymers prior to self-assembly, chemical crosslinking is a suitable strategy for the stabilization of the resulting micelles.^72^,^73^ These crosslinking methods can target the core, corona, or core-corona interface and can involve the use of either additives that link polymer chains by the coupling of functional groups, or block copolymers with coupling sites already present. Due to the demand for improved crosslinking methods to stabilize micelle morphologies, a variety of covalent crosslinking reactions have been developed.^72^,^74^ By the selective localization of functional or crosslinkable groups, micelle structures can be tuned to target particular applications. For example, reversible disulfide crosslinks within the coronas of block copolymer micelles have been used for the controlled release of bioactive molecules from within the core.^75^

To prepare well-defined micelles, the starting block copolymers are first synthesized, isolated and purified. Relatively high dilution is generally used to avoid undesired aggregation of the polymers during self-assembly and to obtain the target micelle morphology in high yields. For the simple and scalable preparation of block copolymer nanostructures, improved and alternative preparative strategies are desirable. Polymerization-induced self-assembly (PISA), which involves the *in situ* self-assembly of block copolymers during polymerization, has recently emerged as an interesting method for the preparation of large quantities of well-defined micelles.^76^,^77^ This approach would be particularly useful if it can be successfully applied to the large scale preparation of “patchy” block copolymer micelles for use in materials applications.

**Fig. 7:**
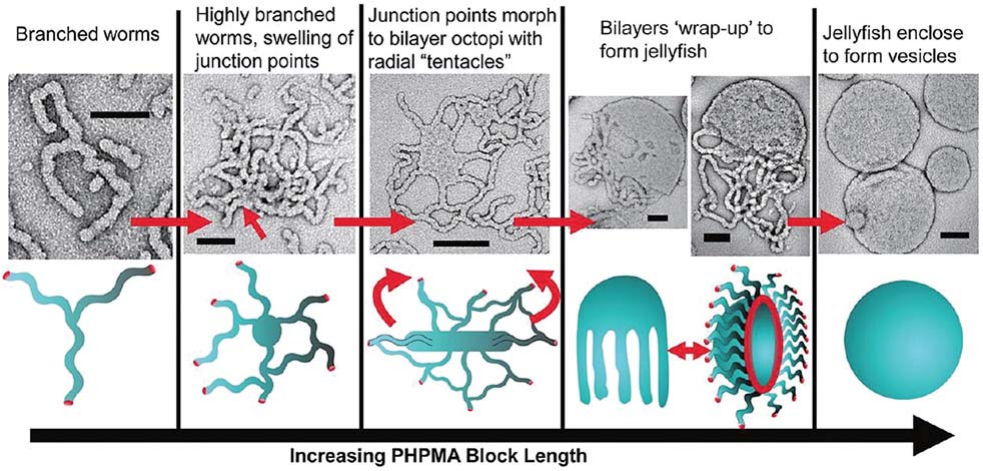
Preparation of non-spherical micelles by RAFT-mediated aqueous dispersion polymerization. Schematic representation and corresponding TEM images of a proposed mechanism for the polymerization-induced worm-to-vesicle transformation during the synthesis of PGMA_47_-*b*-PHPMA_200_ (PHPMA = poly(2-hydroxypropyl methacrylate)). Scale bars correspond to 100 nm. Reproduced with permission from ref. 85. Copyright 2012 American Chemical Society.

Research in the area of PISA has mainly focussed on the use of controlled radical polymerization, however, living anionic^78^ and ROMP^79^ methods have also been used. Due to the development of controlled radical polymerization methods, reactions can be performed in almost any solvent including water. PISA usually relies on the use of a pre-made and soluble macroinitiator. Depending on whether the macroinitiator reacts with a soluble or insoluble monomer, the process is classified as a dispersion polymerization or emulsion polymerization, respectively.^80^ As the chain length of the polymerizing block increases it becomes solvophobic, triggering self-assembly. The first nanostructures to form are always spherical micelles, as the soluble block has a higher degree of polymerization than the solvophobic block during the early stages of polymerization. As the polymerization continues within the micelles, non-spherical morphologies can form from the spherical micelles as the block ratio of the copolymer changes. This sphere to non-sphere transition was first observed indirectly by small-angle neutron scattering for the anionic polymerization of isoprene and styrene.^81^ Shortly thereafter, non-spherical morphologies were prepared by controlled radical polymerizations under both aqueous dispersion^82^,^83^ and emulsion conditions.^84^ For example, RAFT-mediated aqueous dispersion polymerizations have been used to prepare non-spherical morphologies. By monitoring the polymerization of 2-hydroxypropyl methacrylate initiated by a poly(glycerol monomethacrylate) (PGMA) macroinitiator, intermediate sphere-to-worm and worm-to-vesicle structures were observed by TEM (Fig. 7).^85^

### 4.2 Hierarchical self-assembly of block copolymer micelles

**Fig. 8:**
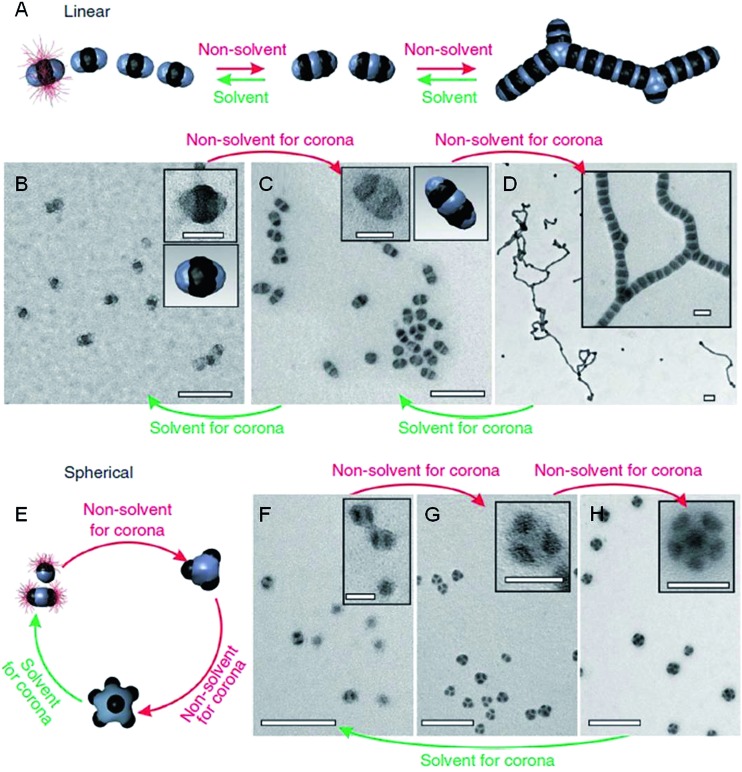
Schematic representation and corresponding OsO_4_ or RuO_4_ stained TEM images of the solvent-driven hierarchical self-assembly of multicompartment micelles. (A) Schematic of reversible colloidal polymerization. The red corona chains emerge from the black compartments, but are mostly omitted for clarity. (B) ‘Hamburger’ subunits in acetone/isopropanol (90/10). (C) ‘Double burgers’ formed by two ‘hamburgers’ in acetone/isopropanol (80/20). (D) Polymerization of ‘double burgers’ in acetone/isopropanol (50/50). (E) Schematic for the reversible exchange of subunits into spherical multicompartment micelles. (F) ‘Hamburgers’ merge into ‘clovers’ (G) and ‘footballs’ (H) triggered by reduction of solvent quality for the corona (addition of isopropanol from 90/10 to 60/40 to 50/50). Subunits reappear upon the expansion of the corona (addition of acetone from 50/50 to 90/10). Scale bars correspond to 200 nm and 50 nm in insets. Reproduced with permission from ref. 88.

Previous work has shown that “patchy” micelles, prepared in solution from triblock copolymers, undergo further self-assembly under kinetic control to afford long 1D multicompartment nanostructures.^86^,^87^ More recently, a series of increasingly complex multicompartment micelles were prepared by the stepwise solution processing and kinetic trapping using triblock copolymers. These “patchy” micelles, with different sizes and valencies, could be used as discrete building blocks for further self-assembly. This was induced by the introduction of a poor solvent for coronal patches on the micelles, and afforded chains, branched structures, clusters (Fig. 8) and two-dimensional lattices.^88^,^89^

**Fig. 9:**
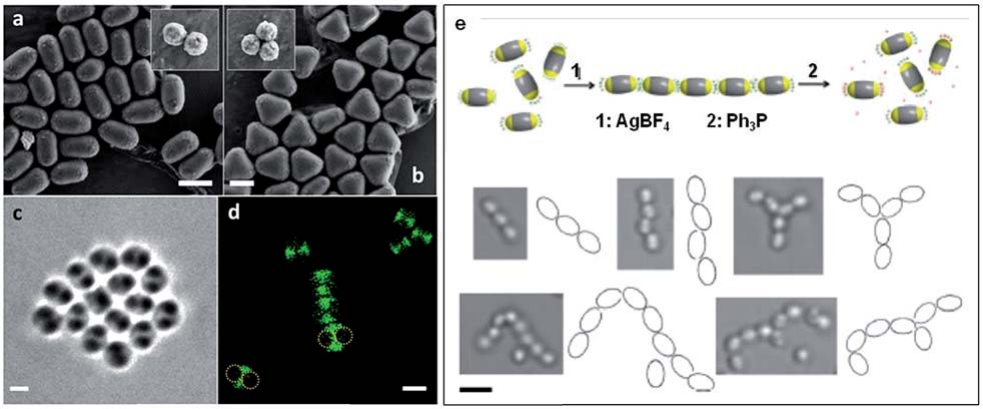
Self-assembly of “patchy” particles *via* metal-coordination. SEM images of (a) two- and (b) three-patch particles (insets show the original clusters). (c) Bright-field image of a two-patch particle in THF, showing the contrast between the patch and antipatch parts. (d) Confocal fluorescence microscopy images of two-patch particles. The anti-patch parts are fluorescently labelled and shown as green. (e) Schematic representation of the triggered self-assembly and disassembly of two-patch particles and accompanying optical microscopy images of the chain structures formed. Scale bars corresponds to 2 μm (a–d) and 3 μm (e). Reproduced with permission from ref. 90. Copyright 2013 American Chemical Society.

As the sizes and patches of the multicompartment micelles can be tuned, the self-assembly process can be controlled. This versatile approach represents a very promising strategy to prepare an almost unlimited variety of hierarchical materials. Moreover, block copolymers can be used to introduce a variety of functional groups with which to direct self-assembly. For example, recent work has shown that “patchy” polystyrene/triblock copolymer-based particles functionalized with metal coordination-based recognition units can undergo coordination-driven self-assembly to afford short chain-like structures (Fig. 9).^90^

Crystallization-driven self-assembly (CDSA) refers to the solution phase behaviour of block copolymers in a selective solvent where, in addition to solvophobic interactions, crystallization of the core-forming block influences the self-assembly process. Core crystallization generally results in a preference for the formation of low curvature micelle morphologies such as cylinders and platelets. A remarkable feature demonstrated for several crystalline-coil block copolymer systems is that the exposed crystalline faces at the core termini of pre-existing micelles remain active to the further growth on the addition of further block copolymer unimer (molecularly dissolved block copolymer). Referred to as living CDSA, this seeded growth process is analogous to a living covalent chain-growth polymerization and can be used to access monodisperse micelles with lengths determined by the unimer-to-seed ratio.^21^ Living CDSA has emerged as a versatile method to prepare block comicelles,^91^ which possess a segmented core and/or corona structure.^19^,^20^ Complex multicompartment micelles are readily prepared by the sequential addition of different block copolymers^92^–^95^ or blends thereof.^96^ In principle, any block copolymer with a crystallizable core-forming block and appropriate block ratio might be expected to undergo living CDSA in selective solvents to afford well-defined cylindrical/fiberlike or platelet morphologies.^97^–^103^ Due to the enthalpy of crystallization of the core-forming block and the resultant kinetic trapping of the resulting micelles, the CMC is effectively zero, and can even be regarded as a meaningless parameter for these non-equilibrium systems.^104^

**Fig. 10:**
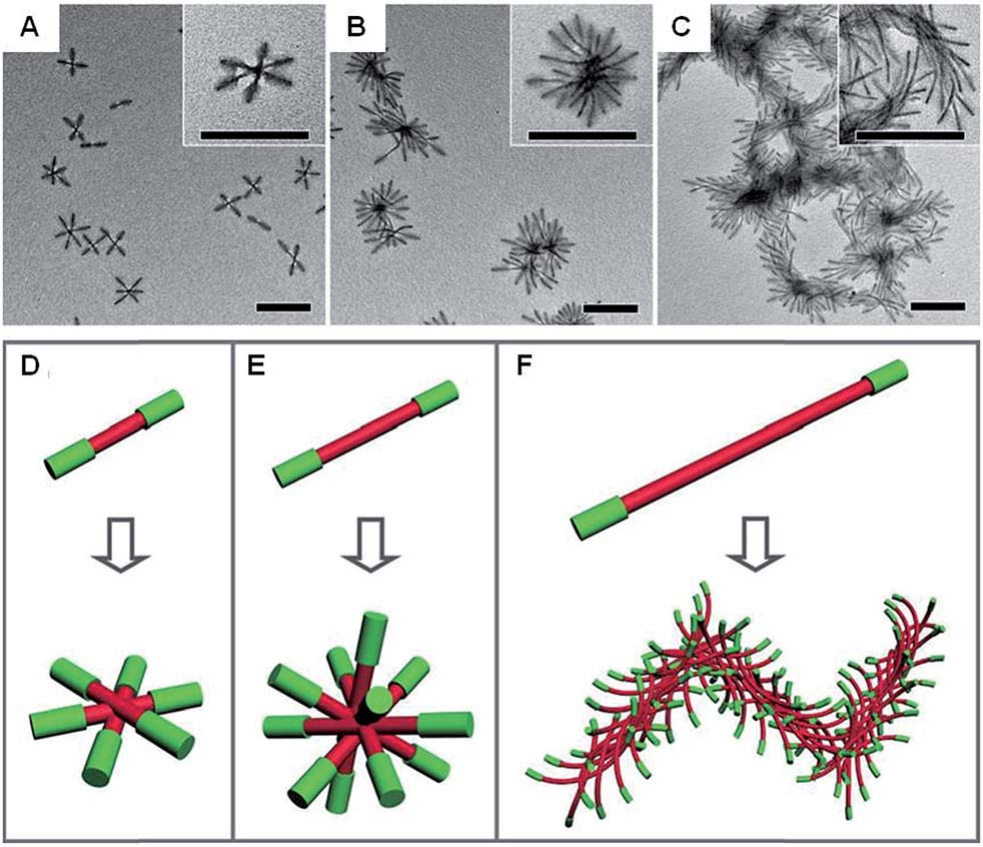
Schematic representations and corresponding TEM images of the hierarchical self-assembly of amphiphilic B–A–B triblock comicelles. M(PFS-*b*-P2VP)-*b*-M(PFS-*b*-PDMS)-*b*-M(PFS-*b*-P2VP) (P2VP = poly(2-vinylpyridine)) with B block lengths of 90 nm and different A block lengths of 110 (A), 260 (B) and 505 nm (C) after dialysis from hexane/isopropanol against pure isopropanol. (D–F) Schematics of the supermicelles formed. For simplicity, the A and B blocks are represented as red and green cylinders, respectively. Scale bars correspond to 500 nm. Reproduced with permission from ref. 106.

Anisotropic block copolymer micelles can be considered as building blocks for further self-assembly. For example, cylindrical multicompartment comicelles prepared by living CDSA are analogous to multiblock copolymers in that they can undergo further solution self-assembly to afford supermicellar structures. On addition of a selective solvent for one of the blocks, amphiphilic A–B–C block comicelles have been shown to undergo further hierarchical self-assembly to afford supermicelles analogous to traditional spherical block copolymer micelles, but on a larger length scale.^105^ When amphiphilic B–A–B triblock comicelles are used instead as the building blocks, a range of different supermicelle morphologies can be prepared. By changing the length of the A block in the starting micelles, the number of aggregated micelles can be tuned to form assemblies ranging from spherical supermicelles to elongated structures (Fig. 10).^106^

**Fig. 11:**
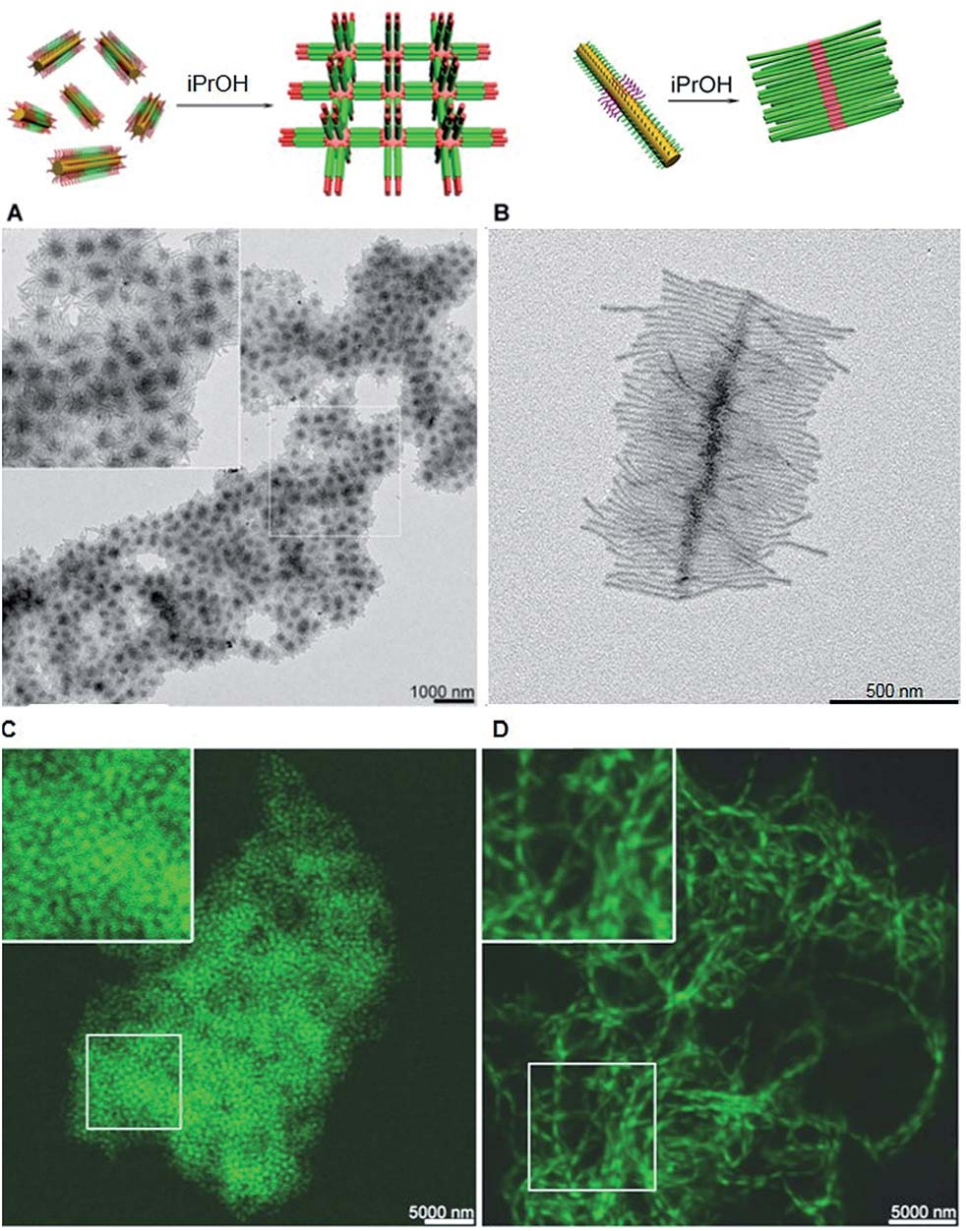
Supermicelles and superlattices by stacking of A–B–A and B–A–B triblock comicelles (A = M(PFS-*b*-P2VP) and B = M(PFS-*b*-PDMS)). (A) TEM images of a 3D superlattice formed by A_105 nm_–B_160 nm_–A_105 nm_ triblock comicelles. (B) TEM images of a cylindrical brush-like supermicelle formed by B_340 nm_–A_35 nm_–B_340 nm_ triblock comicelles in iPrOH. (C) Confocal fluorescence microscopy images of a 3D superlattice formed by A/F_300 nm_–B_570 nm_–A/F_300 nm_ triblock comicelles in iPrOH (F = hydrophobic fluorescent block copolymer). (D) Confocal fluorescence microscopy images of a 1D superlattice formed by A/F_745 nm_–B_570 nm_–A/F_745 nm_ triblock comicelles in iPrOH. Reproduced with permission from ref. 107.

The structure of supermicelle assemblies can be controlled by altering the functionality and dimensions of the starting block comicelles, and adjusting the solution processing protocol used to induce self-assembly. By these techniques, 3D and 1D superlattices have been prepared through the selective side-to-side or end-to-end stacking of A–B–A triblock comicelles (Fig. 11A and B).^107^ As the chemical functionality within the micelle coronas remains unchanged throughout assembly, this represents a practical method with which to transfer function from the molecular level to the resulting supermicelles. For example, by selectively incorporating a fluorescent block copolymer into the A blocks within the micelle subunits, fluorescent 3D and 1D superlattices were prepared (Fig. 11C and D).

## 5 Conclusions

Multicompartment or “patchy” colloidal nanoparticles are finding increasingly widespread use as building blocks for the preparation of complex and functional structures exhibiting one-, two-, or three-dimensional hierarchical order. An advantage of this “polymerization” approach is that the building blocks can be used to transfer functionality from the molecular to the nanometer and micron size regimes. Furthermore, as the shape and surface functionality of the subunits can be tuned during their synthesis, an enormous variety of potential “monomeric” building blocks is possible, varying from hard to soft matter, and also including hybrid systems. In particular, the development of improved methods for controlling the self-assembly of block copolymers has permitted the design of increasingly tailored micelles bearing surface patches of defined chemical functionality. This has allowed the formation of a variety of new materials with unprecedented and complex hierarchical architectures.

Although the vast majority of the “polymerizations” reported to date for “patchy” colloidal particle building blocks probably involve isodesmic, step-growth type mechanisms, the broader realization of cooperative, nucleation–elongation/chain-growth processes, and even living systems, is an especially desirable target for the future. Another fascinating area for further study is related to the question of whether the resulting supracolloidal assemblies exist in a static or dynamic state. Kinetic trapping has been suggested for the cases of cylindrical supermicelles,^107^ and also for supracolloidal polymers formed by spherical diblock copolymer micelles,^108^ which are able to form segmented architectures.

Clearly much needs to be done in terms of performing in depth studies that target increased fundamental understanding of these unusual self-assembling systems. Ultimately however, research in this field is driven by the expectation that new functional materials will be accessible with degrees of hierarchical complexity that are impressive in comparison to those already prevalent in Nature.
